# Supplementary material for: Development of a red fluorescent protein-based cGMP indicator applicable for live-cell imaging
Source: Commun Biol. 2022 Sep 5;5:833. doi: 10.1038/s42003-022-03790-2 (PMC9445041; doi:10.1038/s42003-022-03790-2)
Supplement: Supplementary file 2 — Supplementary Information [file 42003_2022_3790_MOESM2_ESM.pdf]

# Supplementary Information

# Development of a red fluorescent protein-based cGMP indicator applicable for live-cell imaging

Mai Takizawa[1, #], Yuri Osuga[1, #], Rika Ishida[1, #], Marie Mita[1, 3], Kazuki Harada[1], Hiroshi Ueda[2], Tetsuya Kitaguchi[2 *], and Takashi Tsuboi[1 *]

[1]Department of Life Sciences, Graduate School of Arts and Sciences, The University of Tokyo, 3-8-1 Komaba, Meguro, Tokyo 153-8902, Japan,

[2]Laboratory for Chemistry and Life Science, Institute of Innovative Research, Tokyo Institute of Technology, 4259 Nagatsuta-cho, Midori-ku, Yokohama, Kanagawa, 226-8503, Japan.

[3] Present address: Biomedical Research Institute, National Institute of Advanced Industrial Science and Technology, 1-8-31 Midorigaoka, Ikeda, Osaka 563-8577 Japan.

[#] These authors contributed equally

Corresponding authors:

*Tetsuya Kitaguchi, Ph.D. E-mail: kitaguct-gfp@umin.ac.jp

*Takashi Tsuboi, Ph.D. E-mail: takatsuboi@bio.c.u-tokyo.ac.jp

**Supplementary Materials and Methods**

**Investigation of reversibility to cGMP in the responses of Red cGull *in vitro***

Emission spectra of purified Red cGull protein were measured in the absence or presence of 0.01 or 0.1 μM cGMP, as described in the main text. Samples with 0.1 μM cGMP were subsequently diluted 10-fold in (A) HEPES or (B) HEPES with 0.1 μM cGMP. After measuring the emission spectra in (A) and (B), the relative fluorescence intensity (FI) produced by diluting cGMP from 0.1 to 0.01 μM cGMP was calculated from $(F_{0.1uM\ cGMP} \times F_A) / (F_0 \times F_B)$.

**Response kinetics of Red cGull to cGMP *in vitro***

Fluorescence intensity of purified Red cGull protein was measured at 595 nm in the fluorescence spectrophotometer every 0.5 s. Basal fluorescence intensity, normalised to 1, was calculated as the average fluorescence intensity during the 10 s immediately before the application of cGMP.

| Gene | Direction | Primer sequences (5’ > 3’) |
|---|---|---|
| *sGcα1*<br><br>(RefSeq ID: NM_021896.6; Gene ID: 60596) | F | GTCATCACGATGCTCAACGC |
| | R | GGGTGTCACTCTCTCTGTGC |
| *sGcα2*<br>(RefSeq ID: NM_001033322.2; Gene ID: 234889), | F | AGGGTCAACCTGGACTCACT |
| | R | CTGCATCCCTATAACCAATAACTTG |
| *sGcβ1*<br>(RefSeq ID: NM_017469.4; Gene ID: 54195), | F | CTGCCAGGAGTCTGGCTATG |
| | R | ATCGGTGCACCTGAAGGAAG |
| *sGcβ2*<br>(RefSeq ID: NM_172810.3; Gene ID 239134), | F | GAATGCCACCGAGGTTGAGA |
| | R | TAGACTTGCTGCTGGTGGTG |
| *Casr*<br>(RefSeq ID: NM_013803; Gene ID: 12374) | F | ACACCTGCTTACCCGGAAGA |
| | R | GTAGGTGTTTCAGGACCTGCC |
| *Gprc6a*<br>(RefSeq ID: NM_153071.1; Gene ID: 210198) | F | CAGCCATGGGAGCTACTTGC |
| | R | AAATCCCCGTGGGCATCAAA |
| *Gapdh*<br>(RefSeq ID: NM_001289726.1; Gene ID 14433), | F | GGAAGGGCTCATGACCACAG |
| | R | ACCAGTGGATGCAGGGATGA |

**Supplementary Table 1** (Related to Fig. 5a, Supplementary Fig.7a ). **List of primer sequences used for real-time PCR analysis**

F: Forward primer, R: Reverse primer.

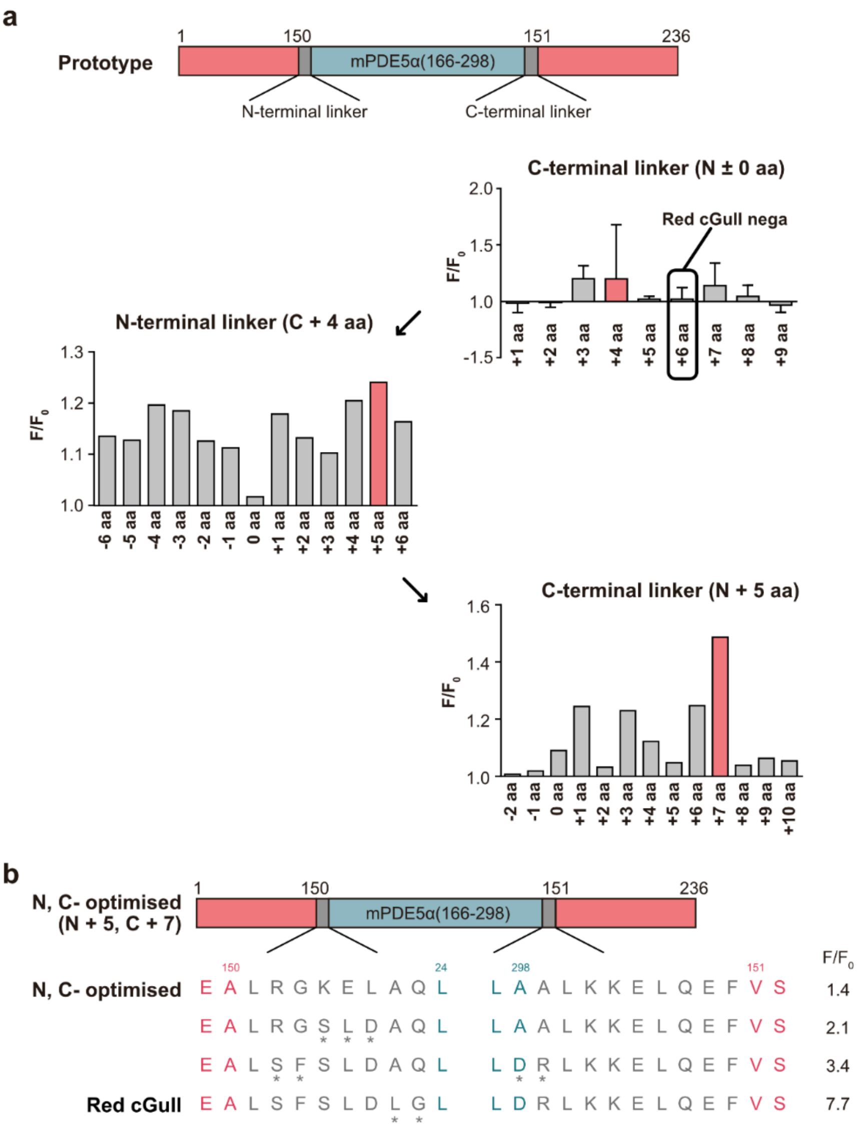


**Supplementary Fig. 1 (Related to Fig. 1). Linker optimisation processes for Red cGull.** (a) Schematic representations (top) and results of linker optimisation for the various candidates with different linkers in the C- and N-terminus (bottom). The first C-terminal linker optimisation was repeated more than four times because of small changes in fluorescence and variance among the colonies of transformed *E. coli* (Data are means + or - standard deviation). Fluorescence responses to 100 μM of cGMP were compared; the greatest response (N + 5, C + 7) was used as the template for site-directed random mutagenesis. The dynamic range ($F/F_0$ if the candidate showed the turn-on type or $F_0/F$ if the candidate showed the turn-off type) is shown in bar graphs. Prototype C + 6 was utilised as Red cGull nega. (b) Results of site-directed random mutagenesis into the template with the optimal linker length (N + 5, C + 7). Asterisks represent the amino acids at which mutations were introduced. Dynamic ranges in response to 100 μM cGMP ($F/F_0$) were calculated and the resultant construct with the highest $F/F_0$ was named "Red cGull."

Red cGull

MAIIKEFMRFKVHMEGSVNGHEFEIEGEGEGRPYEAFQTAKLKVTKGGPLPFAWDILSPQ
FMYGSKAYIKHPADIPDYFKLSFPEGFRWERVMNFEDGGIIHVNQDSSLQDGVFIYKVKL
RGTNFPPDGPVMQKKTMGWEALSFSLDLGLHIHGLISADRYSLFLVCEDSSKDKFLISRL
FDVAEGSTLEEASNNCIRLEWNKGIVGHVAAFGEPLNIKDAYEDPRFNAEVDQITGYKTQ
SILCMPIKNHREEVVGVAQAINKKSGNGGTFTEKDEKDFAAYLDRLKKELQEFVSERMYP
EDGALKSEIKKGLRLKDGGHYAAEVKTTYKAKKPVQLPGAYIVDIKLDIVSHNEDYTIVE
QCERAEGRHSTGGMDELYK

**Supplementary Fig. 2 (Related to Fig. 1). Amino acid sequences of Red cGull.**

The colours of the text correspond to those shown in Supplementary Fig. 1.

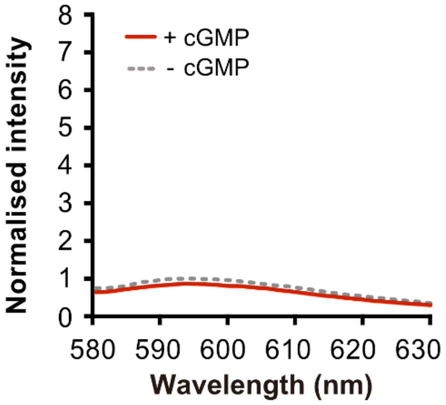


**Supplementary Fig. 3 (Related to Fig. 2). Emission spectra of Red cGull nega.**

The emission spectra of 5 μM Red cGull nega in the in the presence (solid line) and absence (dashed line) of 10 μM of cGMP. The FI was normalised to the maximum FI in the absence of cGMP.

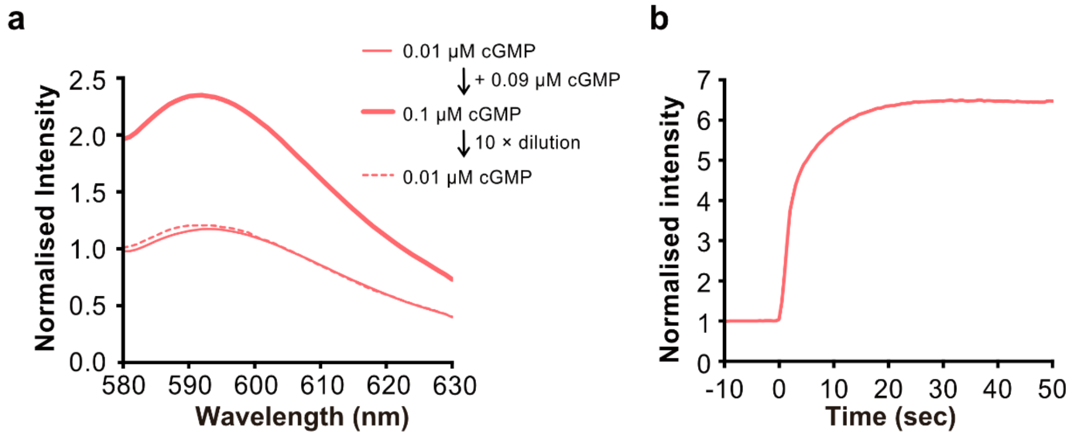


**Supplementary Fig. 4 (Related to Fig. 2). Reversibility and kinetics of Red cGull.**

(a) Emission spectra of purified Red cGull protein in the presence of 0.01 μM cGMP (solid, magenta line), 0.1 μM cGMP (solid, bold magenta line), and 0.01 μM cGMP after the dilution of 0.1 μM cGMP (dashed, magenta line). The peak of fluorescence intensity (FI) in the absence of cGMP was normalised to 1. (b) Time courses of FI for Red cGull protein upon the application of cGMP.

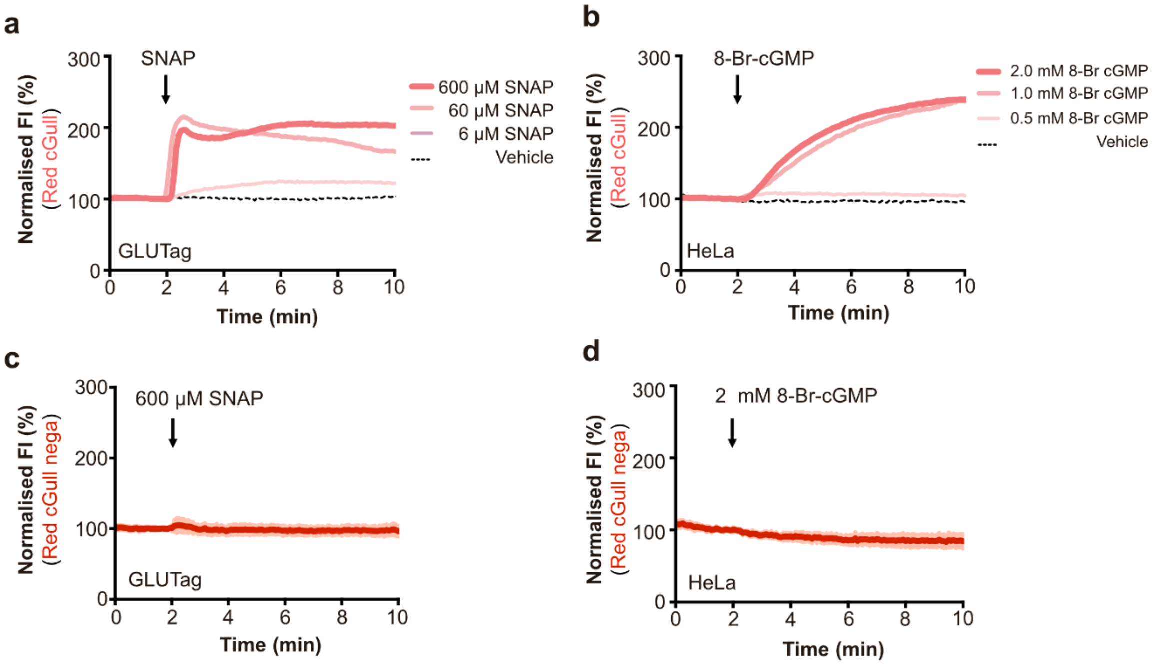


**Supplementary Fig. 5 (Related to Fig. 3) Visualisation of intracellular cGMP dynamics by Red cGull and Red cGull nega.**

(a) Time course of FI of GLUTag cells expressing Red cGull during the application of vehicle or SNAP. Data are shown as means (Vehicle: n = 19 cells from three independent experiments, 6 μM SNAP: n = 31 cells from five independent experiments, 60 μM SNAP: n = 38 cells from five independent experiments, 600 μM SNAP: same data as Figure3a). (b) Time course of FI of HeLa cells expressing Red cGull during the application of 8-Br-cGMP. Data are shown as means (Vehicle: n = 16 cells from three independent experiments, 0.5 mM 8-Br-cGMP: n = 13 cells from three experiments, 1 mM 8-Br-cGMP: n = 15 cells from three independent experiments, 2 mM 8-Br-cGMP: same data as Figure 3b). (c) Time course of FI of GLUTag cells expressing Red cGull nega during the application of 600 μM SNAP. Data are means (line) ± standard deviation (s.d.) (n = 14 cells from three independent experiments). (d) Time course of FI of HeLa cells expressing Red cGull nega during the application of 2 mM 8-Br-cGMP. Data are means (line) ± s.d. (n = 19 cells from three independent experiments).

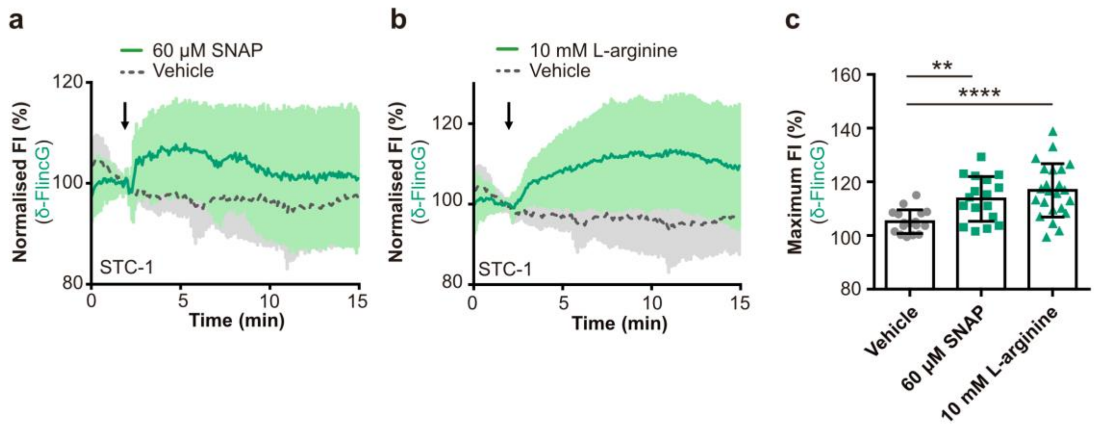


**Supplementary Fig. 6 (Related to Fig. 3, 5) Visualisation of intracellular cGMP dynamics by δ-FlincG.**

(a) Time course of FI of STC-1 cells expressing δ-FlincG during the application of vehicle or 60 μM SNAP. Data are means (line) ± standard deviation (s.d.) (shaded area) (Vehicle and 60 μM SNAP: n = 17 cells from three independent experiments). (b) Time course of FI of STC-1 cells expressing δ-FlincG during the application of vehicle or 10 mM L-arginine. Data are means (line) ± s.d. (shaded area) (Vehicle: same data as (a), 10 mM L-arginine: n = 23 cells from three independent experiments). (c) Maximum FIs of (a) and (b). Data are means ± s.d. One-way ANOVA with Dunnett's multiple comparisons test. From left to right: $p = 0.0071$ (**), $p < 0.0001$ (****).

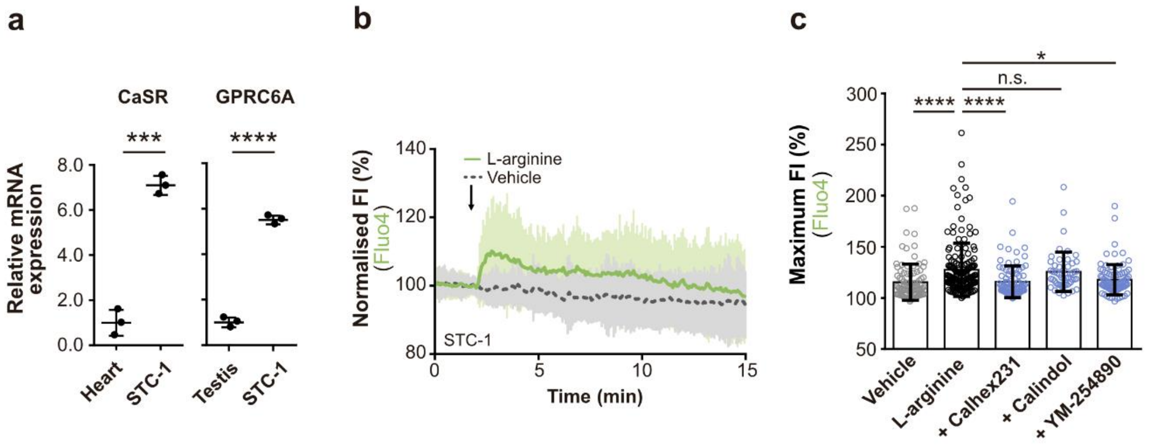


**Supplementary Fig. 7 L-arginine potentiates $Ca^{2+}$ mobilisation in STC-1 cells.**

(a) Relative mRNA expression level of CaSR and GPRC6A in STC-1 cells measured using real-time PCR. GAPDH was used as the internal control. Data are means ± standard deviation (s.d.) from three independent experiments. Welch's *t* test. From left to right: $p = 0.0002$ (***), $p < 0.0001$ (****) (two-tailed). (b) Time course of the FI of Fluo4-loaded STC-1 cells during the application of 10 mM L-arginine (solid line) or vehicle (dashed line). Data are means (line) ± s.d. (shaded area) (vehicle: n = 94 cells from six independent experiments, L-arginine: n = 163 cells from 10 independent experiments). (c) Maximum FI of Fluo4 during the application of 10 mM L-arginine in the presence of 400 nM of Calhex231 (a CaSR antagonist), 1 µM of Calindol (a GPRC6A antagonist), or 250 nM of YM-254890 (a Gq protein inhibitor). Data are means ± s.d. (Vehicle and L-arginine: same data from (b), Calhex231: n = 88 cells from five independent experiments, + Calindol: n = 51 cells from four independent experiments, + YM-254890: n = 91 cells from six independent experiments). Kruskal-Wallis test with Dunn's multiple comparison. From left to right: $p < 0.0001$ (****), $p < 0.0001$ (****), $p > 0.9999$ (n.s.), $p = 0.0257$ (*).

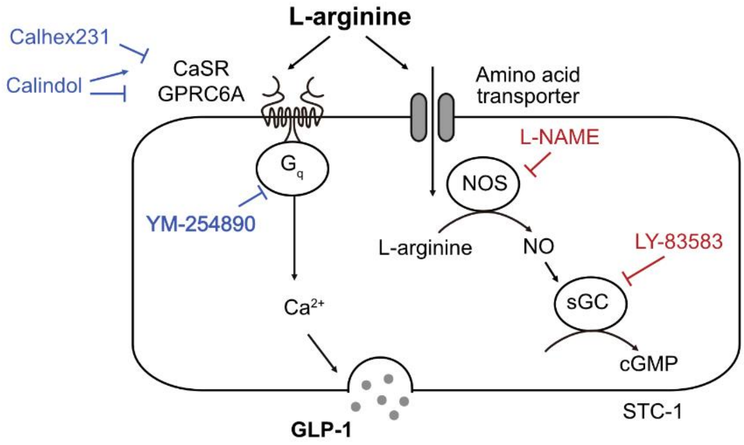


**Supplementary Fig. 8 Schematic model of L-arginine-induced GLP-1 secretion from STC-1 cells.**

cGMP production through activation of the NO/cGMP signalling pathway has little effect on GLP-1 secretion, whereas CaSR plays a role in inducing an increase in the intracellular $Ca^{2+}$ concentration, which in turn promotes GLP-1 secretion. The reagents used in the experiments are also shown in this model.
